# PSYCHOLOGICAL DISORDERS LINKED TO OSTEOPOROSIS IN A POPULATION-BASED COHORT STUDY

**DOI:** 10.1093/geroni/igac059.1774

**Published:** 2022-12-20

**Authors:** Kanya Godde Chrisco, Margaret Gough Courtney, Josephine Roberts, Yadira Quintero

**Affiliations:** University of La Verne, La Verne, California, United States; University of La Verne, La Verne, California, United States; University of La Verne, La Verne, California, United States; University of La Verne, La Verne, California, United States

## Abstract

Psychosocial disorders can stem from or have profound effects on one’s health, having been linked to many negative health outcomes. In this study, we hypothesize psychological disorders are associated with a higher risk osteoporosis diagnosis. Self-reported information from years 2012-2016 of the public-use, longitudinal cohort-based Health and Retirement Study, was evaluated from 11,716 American respondents aged 50-90 years old. The odds of scores on the Center for Epidemiological Studies Depression (CESD) scale, and broader psychological disorders (emotional, nervous, psychiatric) on osteoporosis diagnosis (outcome), were estimated with a logistic regression using survey weights, while controlling for sex, logged age, education level, race/ethnicity, family structure during childhood (number of adults), having thyroid disease, allostatic load, and body weight. A McFadden’s R2 (0.18) shows the model fits relatively well. The results demonstrate that as CESD score goes up, there is a 10% increase in odds (OR = 1.1, P < 0.001) of an osteoporosis diagnosis. Similarly, if a respondent reported a doctor told them they had other psychological disorders, the odds of an osteoporosis diagnosis increased by 52% (OR = 1.52, P < 0.001). It is unknown whether the components of broader psychological disorders are caused by decreased quality of life and/or other limitations from osteoporosis or if they contribute to bone health changes in this sample, or both. However, as CESD is a short-term measure (reflecting on the week prior) it is deduced to be as a result of a decreased quality of life associated with some cases of osteoporosis.

